# Cocaine

**Published:** 1885-01

**Authors:** 


### COCAINE.

Never within our knowledgs has the discovery of any new drug aroused the attention of the medical profession so thoroughly as has that of the properties of the Hydrochlorate of Cocaine. In all the departments of medicine it has been tried as a local anæsthetic. So general was the demand for it that the price, immediately upon its discovery, rose to an absurd point. Its effect upon mucus tissue is positively astonishing, and hence it has at once taken an important place in the materia medica of ophthalmology, and bids fair to become no less essential to the Gynæcologist. In dentistry its position is not yet fully determined. Some conflicting accounts are given. We have been favored with so many communications regarding it that there is not space to print them all, but the first three received are given in this number.

We have used it to a limited extent, but personal experience does not lead to the conclusion that the dental millennium has prematurely arrived. On all the mucus tissues of the mouth it exerts a charming influence, but as an obtunder of sensitive dentine, in our hands it has not so far proved reliable. We are inclined to the opinion that a strength of at least ten per cent. might prove more effective. It seems probable that hypodermic injections in the neighborhood of nerve trunks, as suggested by Dr. Hepburn in his excellent article in this number, might prove efficacious. In the meantime, as it is now obtainable, it would seem desirable that any operative dentist should make trial of its virtues, as by this means we shall, with the greater certainty and quickness, obtain an exact opinion of its merits in dental practice.

